# Involvement of the NLRC4-Inflammasome in Diabetic Nephropathy

**DOI:** 10.1371/journal.pone.0164135

**Published:** 2016-10-05

**Authors:** Fang Yuan, Ryan Kolb, Gaurav Pandey, Wei Li, Lin Sun, Fuyou Liu, Fayyaz S. Sutterwala, Yinghong Liu, Weizhou Zhang

**Affiliations:** 1 Department of Nephrology, the Second Xiangya Hospital, Research Institute of Nephrology, Central South University, Changsha, Hunan China; 2 Department of Pathology, University of Iowa Carver College of Medicine, Iowa City, IA, United States of America; 3 Interdisciplinary Graduate Program in Immunology, University of Iowa Carver College of Medicine, Iowa City, IA, United States of America; 4 Department of Medicine, Cedars-Sinai Medical Center, Los Angeles, CA, United States of America; 5 Medical Scientist Training Program, University of Iowa Carver College of Medicine, Iowa City, IA, United States of America; 6 Free Radical and Radiation Biology Program, University of Iowa Carver College of Medicine, Iowa City, IA, United States of America; 7 Cancer Genes and Pathway Holden Comprehensive Cancer Center, University of Iowa Carver College of Medicine, Iowa City, IA, United States of America; University of Louisville, UNITED STATES

## Abstract

Diabetic nephropathy (DN) is the leading cause of end-stage kidney disease worldwide but current treatments remain suboptimal. The role of inflammation in DN has only recently been recognized. It has been shown that the NLRP3-inflammasome contributes to DN development by inducing interleukin (IL)-1β processing and secretion. In an effort to understand other IL-1β activating mechanism during DN development, we examined the role of the NLRC4-inflammasome in DN and found that NLRC4 is a parallel mechanism, in addition to the NLRP3-inflammasome, to induce pro-IL-1β processing and activation. We found that the expression of NLRC4 is elevated in DN kidneys. NLRC4-deficiency results in diminished DN disease progression, as manifested by a decrease in blood glucose and albumin excretion, as well as preserved renal histology. We further found that DN kidneys have increased F4/80^+^ macrophages, increased IL-1β production, and other signaling pathways related to kidney pathology such as activation of NF-κB and MAP kinase pathways, all of which were rescued by NLRC4-deficiency. This study demonstrates NLRC4-driven IL-1β production as critical for the progression of DN, which underscores the importance to target this pathway to alleviate this devastating disease.

## Introduction

Diabetic nephropathy (DN) is the leading cause of end-stage kidney disease worldwide but current treatments remain suboptimal. DN is manifested with the early signs of glomerular hyperfiltration, the hypertrophy of both glomeruli and tubules, the increased thickness of glomerular basement membrane (GBM), as well as with the late signs of increased mesangial extracellular matrix and proteinuria, ultimately resulting in glomerulosclerosis and fibrosis. Classic risk factors for DN include high blood glucose, lasting diabetes, blood pressure, obesity and related lipidemia. [1].

Recent studies have identified a connection between inflammation and the development and progression of DN. In particular, macrophages and T cells have been shown to be the effector cell types that contribute to the disease progression in mouse DN model [2, 3]; in human DN patients, there is a positive correlation between the extent of renal inflammatory cells and DN progression [4, 5]. DN disease severity was able to be reversed by the inhibition of inflammatory cell recruitment [6, 7]. As major mediators of pro-inflammatory response from immune cells, cytokines or chemokines are known to be involved in DN [8, 9]. Interleukin (IL) 1β, IL-6, TNF-α etc have been shown to be produced from endothelial, mesangial, glomerular and tubular epithelial cells and are involved in the aggravation of DN [10, 11].

Inflammasome activation, one of the major ways that innate immune cells use to produce IL-1β and IL-18, has been shown to be critical for insulin resistance and diabetes. Inflammasomes are pro-inflammatory protein complex consisting of one of the Nod-like receptors (NLR) including Nlrp1, Nlrp3, Nlrc4 or Pyrin family Aim2, adapter protein ASC/Pycard, and Caspase 1 (Casp-1). Inflammasomes sense different pathogen-associated or danger-associated molecular patterns (PAMPs or DAMPs) and oligomerize these components into a very gigantic and efficient protein complexes, leading to Casp-1 activation and IL-1β and IL-18 production, mainly from innate immune cells [12]. Many recent publications provide new evidence that chronic inflammasome activation, likely from intrinsic DAMPs, is the culprit for many important chronic diseases such as diabetes where low grade inflammation occurs without clear pathogen infection. In particular, NLRP3-inflammasome has been shown to exacerbate the development of type 2 diabetes (T2D) [13] as well as DN progression [14–18]. The role of other inflammasomes in DN is currently unknown.

NLRC4-inflammasome is only known to be activated by bacterial protein products which are specifically mediated by NAIP family members, such as NAIP1 for the type III secretion system (T3SS) needle complex [19], NAIP2 for the T3SS rod protein [20], both NAIP5 and NAIP6 recognizing flagellin [20, 21]. The role of NLRC4-inflammasome in the development of T2D and DN has not been reported. Here we found that NLRC4 expression increased in kidney specimens of DN patients. We found that NLRC4-inflammasome is as critical as NLRP3 for promoting DN progression, indicating that there are multiple IL-1β-activating mechanisms in DN. Our result, together with others, further justified the blockage of the inflammasome/IL-1β signaling to prevent DN progression.

## Materials and Methods

### Morphological analysis of human kidney samples

Human kidney biopsy tissues were obtained from T2D patients (n = 10) of 10–15 years’ duration, and an equal number of normal kidney tissues from nephrectomies of renal hamartoma (n = 6) were recruited as control for the study (the kidney samples were archived retrospectively from 09/2012-12/2013 and we did the experiments in 05/2014). The renal sections were stained with periodic acid schiff (PAS) according to standard procedures. Semiquantitative scoring of glomerular sclerosis in PAS-stained slides was performed using a five-grade method described previously [22]. Section immunohistochemistry was performed using primary antibody against NLRC4 (Sigma-Aldrich), CD68 (Santa Cruz). Antigen signals were detected with polymer detection kit (Biocare medical). The human protocol was approved by the Institutional Human Experimentation Ethics Committee, Second Xiangya Hospital, Central South University (No:S065).

### Animal experimental design

NLRC4-deficient mice (*Nlrc4*^-/-^) on a C57BL/6N genetic background have been previously described [23]; wild-type (WT) C57BL/6N mice were purchased. The type 2 diabetic mouse model was developed using high-fat diet plus streptozotocin (STZ), as described previously [24, 25]. After mice were fed high-fat diet (consisting of 20.5% protein, 36% fat and 35.7% carbohydrate with a total calorific value of 5.49 kcal/gm, Bio-Serv) for 4 weeks, the STZ (100 mg/ kg, dissolved in 10mM citrate buffer, pH 4.2, Sigma) was *i*.*p*. injected into mice. The fasting plasma glucose (FPG) was tested after 12 hrs to select mice with FPG ≥300 mg/dL as a diabetic model. Three groups of mice (n = 6), i) the control group (wild-type mice that were fed with regular food and were injected with a normal saline); ii) the wild-type diabetic group; iii) *Nlrc4*^-/-^ diabetic group. All animals were killed at 8 weeks following STZ administration and kidneys were immediately harvested for protein and RNA extraction, or for histological analyses. This study was carried out in strict accordance with the recommendations in the Guide for the Care and Use of Laboratory Animals of the National Institutes of Health. The animal experimental protocol as described was approved by the Institutional Animal Care and Use Committee (IACUC) of the University of Iowa (ACURF: 5071455). Our institute has institutional assurances including accredited by The Association for Assessment and Accreditation of Laboratory Animal Care, International (AAALAC International) (since November 1994), registered in the United States Department of Agriculture research facility (USDA No. 42-R-0004), as well as the strict following of the PHS Animal Welfare Assurance (A3021-01).

### Blood and urine examination

The blood glucose level, urine samples and the body weight were evaluated every other week for 8 weeks. The blood glucose level was measured in tail vein blood. The urinary creatinine levels and urinary albumin concentration were determined. The results were expressed as the urinary albumin/creatinine ratio (ACR) [25].

### Renal histology and immunohistochemistry

Paraffin embedded mouse kidneys were sectioned and stained with hematoxylin and eosin (H & E) or with PAS. Mean glomerular tuft volume was determined from the glomerular cross-sectional tuft area and mesangial matrix index was determined as described previously [26]. At least 20 glomeruli per mouse were scored from 4–5 mice per group. Immunohistochemistry was performed using anti-F4/80 antibody (Santa Cruz Biotechnology, Santa Cruz, CA, USA) and was detected using the HRPO-polymer (Biocare Medical).

### Kidney tissue processing and flow cytometric analysis of kidney macrophage

Single renal cell suspensions were prepared from WT diabetic, *Nlrc4*^-/-^ diabetic or control mice at 8 weeks after STZ administration. Kidneys were physically cut into small pieces and digested with collagenase type IV (10 μg/ml; Sigma-Aldrich) and DNase I (Sigma-Aldrich) for 1 hour at 37°C. The digested kidney suspension was spun down and added RBC lysis buffer. Then the suspension was filtered through a 40-μm strainer (BD Bioscience, San Diego, CA, USA) and spun down. Single cells were stained with fluorochrome-labelled antibody mixture including anti-CD45, anti-CD11b, anti-CD11c, and anti-F4/80 (eBioscience, San Diego, CA, USA), following by flow cytometric analysis (Accuri C6 Flow Cytometer, BD Bioscience) with the FlowJo software (Tree Star Inc., Ashland, CA, USA).

### IL-1β analysis by Enzyme-linked immunosorbent assay (ELISA)

After lysing kidney tissues, supernatants were collected from each group to detect the level of IL-1β using cytokine ELISA kits (BD Biosciences, San Diego, CA, USA) according to the manufacturer’s instructions.

### Western blot

30μg total tissue protein extracted from mouse kidney was separated on 8–12% SDS-PAGE and electrophoretically transferred to polyvinylidene difluoride (PVDF) membrane pre-activated by methanol in the transferring buffer. Membranes were blocked with 5% skimmed milk for 30min, and were incubated 2-hour with specific primary antibodies at room temperature. Immunoreactive bands were detected using HRP-conjugated goat anti-rabbit or goat anti-mouse IgG as the secondary antibody (1:5000) (Santa Cruz). Primary antibodies included antibodies against Caspase1 p10 (GeneTex), p-JNK, NF-κB p100/52, p-IκBα and tubulin (Cell Signaling).

### Real-time PCR analysis

RNA specimens were purified from mouse kidneys (Qiagen), following by cDNA synthesis using iScript cDNA synthesis kit (BIO-RAD). Real-time PCR (RT-PCR) was performed with a SYBR green master mix (BIO-RAD) using ViiA 7 Real-Time PCR System (Thermo Fisher Scientific). As an internal control, GAPDH levels were quantified in parallel with targeted genes. Normalization and fold change for each of the genes were calculated using the 2^-(ΔΔCt)^ method. Here are the primers used for real-time PCR. *Nlrc4*, forward: 5’- ATCGTCATCACCGTGTGGAG-3’; reverse: 5’- GCCAGACTCGCCTTCAATCA-3’. *Casp-1*, forward: 5’- ACAAGGCACGGGACCTATG-3’; reverse: 5’- TCCCAGTCAGTCCTGGAAATG-3’. *Mcp-1*, forward: 5’- TTAAAAACCTGGATCGGAACCAA-3'; reverse: 5’- GCATTAGCTTCAGATTTACGGGT-3’. *Tgf-β1*, forward: 5’-CAACCCAGGTCCTTCCTAAA-3’; reverse: 5’- GGAGAGCCCTGGATACCAAC-3'. *Ctgf*, forward: 5’- GCTTGGCGATTTTAGGTGTC-3’; reverse: 5’- CAGACTGGAGAAGCAGAGCC-3’. *Tnfα*, forward: 5’- AGGGTCTGGGCCATAGAACT-3'; reverse: 5’- CCACCACGCTCTTCTGTCTAC-3’. *Gapdh*, forward: 5’- TTGATGGCAACAATCTCCAC-3’; reverse: 5’- CGTCCCGTAGACAAAATGGT-3’.

### Statistical analysis

The results are presented as the mean ± SEM. Statistical analysis was performed by ANOVA or by the student's t-test. A P value less than or equal to 0.05 was considered statistically significant.

## Results

### Increased expression of NLRC4 and macrophage infiltration in renal tissues of patients with DN

**Fig 1:**
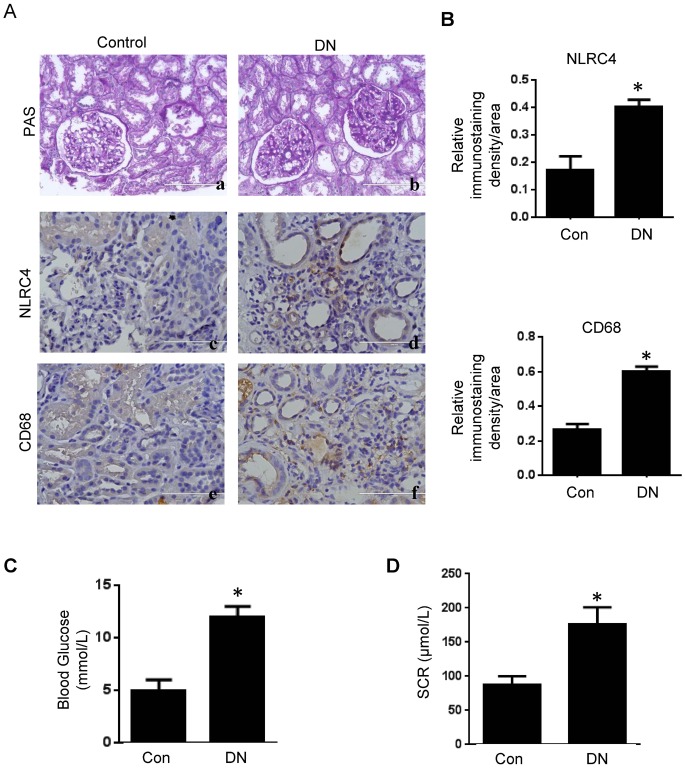
Increased expression of NLRC4 and macrophage infiltration in renal tissues of DN patients. **(A)**: PAS staining showing GBM thickening and mesangial expansion in renal biopsies in DN patients (Aa vs. Ab, Scale Bar: 200 μm). IHC studies revealed increased expression of NLRC4 and CD68 in DN patients (Ac-Af, Scale Bar: 100 μm). **(B)**: Averaged relative intensity for the staining of NLRC4 (top panel) and CD68 (bottom panel) in kidney biopsies of control versus DN patients. **(C and D)**: Blood glucose levels and serum creatinine (SCR) in control and DN patients. Values are means ± s.e.m. Con: control patient; DN: diabetic patient. *P<0.05.

The NLRP3-inflammasome is critically involved in DN progression through the cleavage and activation of IL-1β [14]. Interestingly, while NLRP3-deficiency partially alleviated DN phenotype, pharmacological inhibition of IL-1 signaling with the recombinant IL-1Ra anakinra completely reversed DN phenotype including albuminuria and extracellular matrix accumulation [14]. We thus hypothesized that other IL-1β activating mechanism may also contribute to DN progression. We decided to determine the role of other inflammasomes in DN. Morphological changes in glomerular and tubulointerstitial compartments, including thickening of glomerular basement membrane (GBM) and mesangial expansion, focal glomerulosclerosis and interstitial fibrosis in DN patients, were visualized by PAS staining relative to the normal kidneys. Immunohistochemistry revealed a significantly increased NLRC4 expression in the renal tubules and interstitium of DN patients compared with that of control patients (Fig 1A). Quantitatively, NLRC4 staining intensity exhibited one fold increased in renal tubulointerstitial of DN patients (Fig 1B). We further examined macrophage infiltration in renal tissues using anti-CD68 immunohistochemistry (Fig 1A). CD68 immunostaining score was increased by more than one fold in DN patients (Fig 1B). Also, increased blood glucose and serum creatinine levels were observed in DN patients (Fig 1C and 1D).

### NLRC4-deficiency attenuated renal injury in mouse DN model

**Fig 2:**
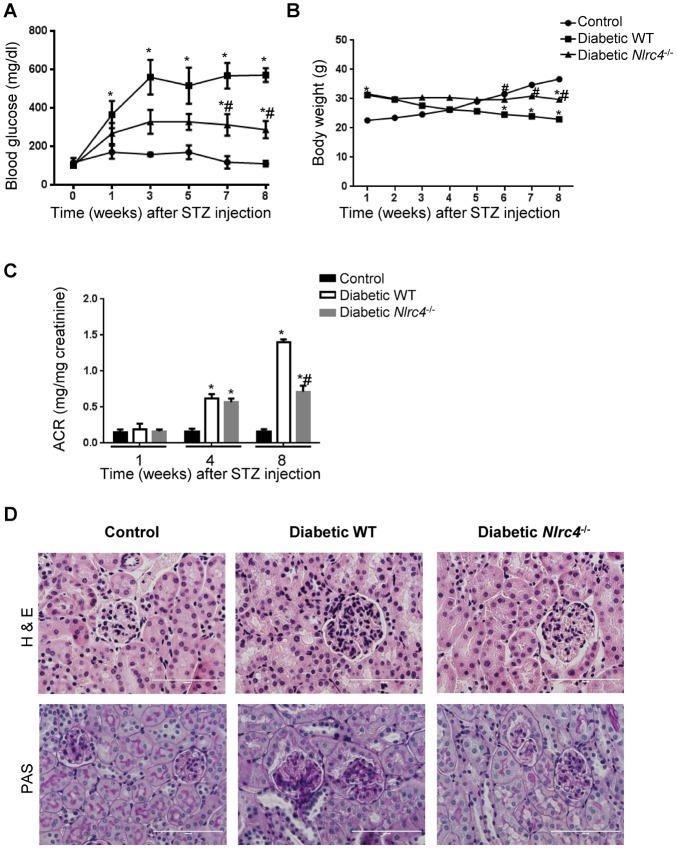
NLRC4-deficiency ameliorates hyperglycemia and renal morphological changes in mice. **A-C**. Mice were fed an HFD for 4 weeks then injected with STZ and monitored for 8 weeks. Blood glucose levels (**A**), body weight changes (**B**), and urinary albumin to creatinine ratio (ACR) (**C**) at different time pointes after STZ treatment were measured from normal WT, diabetic WT and diabetic *Nlrc4*^-/-^ mice. **D**. Mice were killed 8 weeks after STZ treatment and the kidneys were harvested. Kidney sections were stained with hematoxylin and Eosin staining (H&E) and PAS staining (magnification×400). *, *P*<0.05 vs. control WT; #, *P* <0.05 vs. diabetic WT. n = 6 for all these experiments. Scale bar: 100 μm.

We used an obesity-relevant type 2 diabetes (T2D) model, by feeding the mice with high-fat diet (HFD) for 4 weeks following with STZ while maintaining HFD for another 8 weeks. This is a valid model for T2D as many T2D patients ultimately develop late stage β-cell destruction when the most severe DN develops [27]. The blood glucose level rose both in wild-type (WT) and *Nlrc4*^-/-^mice one week after STZ treatment (Fig 2A) and hyperglycemia persisted during the 8-week period (Fig 2A). The mice exhibited reduction in body weight during this time (Fig 2B). The diabetic *Nlrc4*^-/-^mice had significantly lower blood glucose levels than the diabetic WT mice (P<0.05) (Fig 2A) and had stable body weight relative to the diabetic WT mice (Fig 2B). As expected, both WT and *Nlrc4*^-/-^ mice developed albuminuria (expressed as urinary albumin to creatinine ratio (ACR)), and the severity increased with time (Fig 2C), but *Nlrc4*^-/-^ mice tended to have lower urinary albumin excretion than WT mice eight weeks after STZ treatment.

Histological assessment of renal tissues further revealed that the *Nlrc4*^-/-^mice developed less severe renal pathology including decreased glomerular hypertrophy, mesangial expansion and basement membrane thickening (Fig 2D). In particular, T2D WT mice exhibited significantly increased kidney/body weight ratio relative to the WT mice (KW/BW, 31.8±7.7 x 10^−3^ in T2D vs. 6.7 ±0.5 x 10^−3^ in control), which was partially but significantly rescued by NLRC4-deficiency (18.6±9.4 x 10^−3^, P < 0.05 relative to T2D WT mice) (Table 1). The increased glomerular volume and mesangial matrix index from WT T2D mice were also partially rescued by NLRC4-deficiency, with glomerular volume decreased from 9.8±1.3 to 7.4 ±0.9 x 10^5^ (μm^3^) and mesangial matrix index from 42.4±7.9 to 39.5±4.4 x 10^−2^ (Table 1). These data suggested that NLRC4 is critical for DN severity in the T2D model.

**Table 1:** Quantitative morphological analysis in mouse kidneys.

Group	N	KW/BW (x 10^−3^)	Glomerular Volume (x 10^5^)	Mesangial Matrix Index (x 10^−2^)
Control	6	6.7 ±0.5	4.5±0.4	33.3 ±6.2
Diabetic WT	6	31.8±7.7*	9.8±1.3*	42.4±7.9*
Diabetic *Nlrc4*^-/-^	6	18.6±9.4#	7.4 ±0.9*#	39.5±4.4*#

Glomerular volume, mesangial matrix index and kidney weight/body weight (KW/BW) were measured. The mesangial matrix index was defined as the proportion of the glomerular tuft occupied by the mesangial matrix area (excluding nuclei). Values are means ± s.e.m.

*P<0.05 vs normal control mice;

^#^*P* <0.05 vs diabetic WT mice.

### NLRC4-deficiency leads to the diminished inflammasome activity in renal tissues from the T2D mice

**Fig 3:**
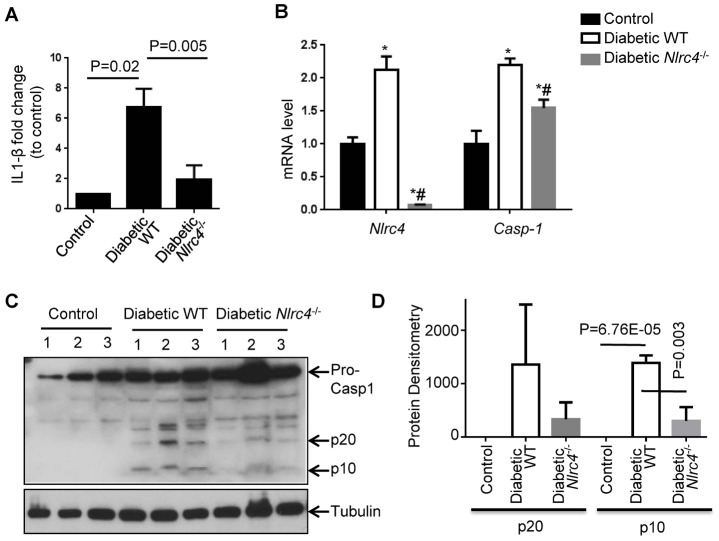
NLRC4-inflammasome activation in the renal tissues of DN mice. **(A)** ELISA analysis of IL-1β levels in kidney tissues from different groups including normal WT, diabetic WT and diabetic *Nlrc4*^-/-^ mice. Values are means ± s.e.m. n = 3. (**B)** The mRNA levels of *Nlrc4* and *Casp-1* in renal tissues from different groups by real time PCR analysis, using *Gapdh* as a reference gene. Values are mean fold change compared to control mice ± s.e.m. *, P<0.05 vs. control WT; #, P <0.05 vs. diabetic WT. n = 3 each group. (**C)** Western blot analyses of cleaved Casp-1 p10 and p20 subunits in renal lysates from different groups. n = 3 each group. (**D)** Protein densitometry analysis of C to show the processing and activation of pro-casp1 into p20 and p10 subunits. Data represents mean protein densitometry in pixels and normalized to the corresponding Tubulin, quantitated by *Image J* ± s.e.m. n = 3.

Nod-like proteins such as NLRP6, NLRP12 and others have different roles under different pathophysiological contexts, including inflammasome-dependent and–independent functions [28]. To examine if NLRC4-inflammasome is activated in the renal tissues from T2D mice, we extracted protein lysates and measured IL-1β level by ELISA and Casp-1 processing by immunoblotting. In agreement with the increased IL-1β level in a mouse model of STZ-induced DN [14], we found that renal tissues from the HFD/STZ-induced DN mice exhibited elevated IL-1β by ELISA (Fig 3A). In addition, we found that the IL-1β is significantly diminished in the renal tissues of the *Nlrc4*^*-/-*^ mice similarly treated with HFD/STZ (Fig 3A). Interestingly, the study showing that NLRP3 was critical for DN progression did not show the NLRP3-dependent IL-1β production [14]. Inflammasome activation involves two steps, including the priming step and the activation step. The priming step involves in the recognition of extrinsic or intrinsic danger signals that further leads to the transcription of inflammasome component. The second activation step is the oligomerization of NLR proteins, the adaptor ASC, and Casp-1 that leads to the processing of pro-Casp-1 into p20 and p10 subunits [29]. We found that renal tissues from DN mice had elevated mRNA of both *Nlrc4* and *Casp-1* (Fig 3B), indicating the priming of NLRC4-inflammasome. While there was little *Nlrc4* mRNA from the renal tissues of *Nlrc4*^-/-^ DN mice, *Casp-1* mRNA was also decreased in the renal tissues of *Nlrc4*^-/-^ DN mice relative to the WT DN mice (Fig 3B), suggesting a positive feedback loop from NLRC4-inflammasome activation to *Casp-1* mRNA expression. In agreement, we did find increased Casp-1 protein levels in the renal tissues from the WT DN mice relative to control WT mice (Fig 3C). Importantly, pro-Casp-1 processing into p20 and p10 subunits, the activating step of inflammasome, was readily seen from renal tissues from the DN WT mice relative to normal WT mice, which is significantly diminished in the renal tissues from the *Nlrc4*^-/-^ DN mice (Fig 3C and 3D). We thus confirmed that NLRC4-inflammasome is activated in renal tissues from the DN mice, which is likely one of the major causes of IL-1β production in the DN renal tissues. It has been shown that inhibiting IL-1β/IL-1R1 axis by anakinra, a recombinant IL-1R1 antagonist approved to treat rheumatoid arthritis, is sufficient to reverse DN pathology in mouse model [14]. Our data, together with others [14], provide a strong rationale to use inflammasome and IL-1β/IL-1R1 inhibitors to treat DN patients.

### NLRC4-deficiency reduces renal macrophage accumulation in T2D mice

**Fig 4:**
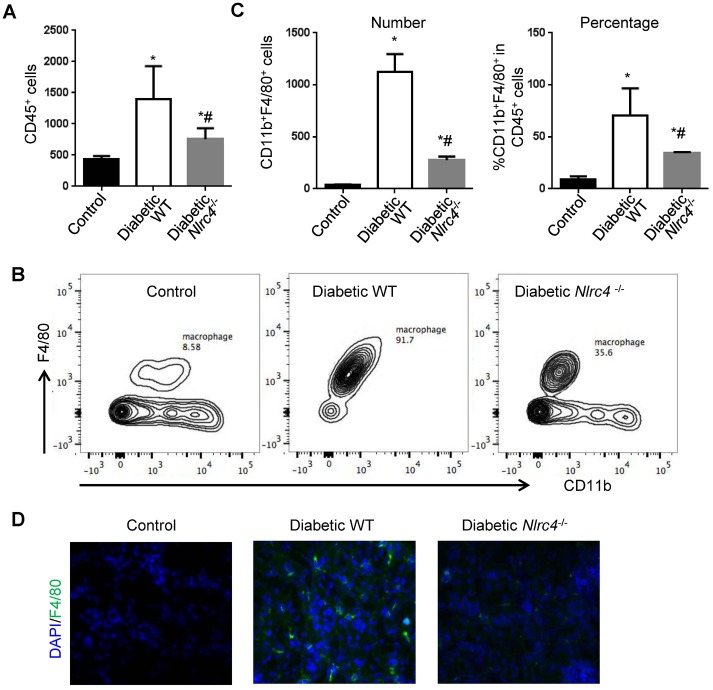
NLRC4-deficiency reduces kidney macrophage accumulation in the DN mice. **(A-C)**. Flow cytometric analysis of CD45^+^ leukocytes (A) and CD11b^+^F4/80^+^ macrophages (B-C) in the renal tissues from different groups. (**B)**. Representative images for flow cytometry for CD11b^+^F4/80^+^ macrophages from different groups. **(C)**. Statistics for total numbers (left panel) and percentages (right panel) of macrophages from B. Values are means ± s.e.m. *, *P*<0.05 vs. control WT; #, *P* <0.05 vs. diabetic WT. **(D)**. Immunofluorescent staining showed for F4/80^+^ macrophages in the renal interstitial space from the indicated mice. n = 6 for all these experiments.

As innate immune cells are the major cellular source for detection of both extrinsic and intrinsic danger signals for inflammasome activation, we reason that the increased NLRC4-inflammasome activation in renal tissues from the DN mice may come from innate immune cells. We examined immune cell infiltration in DN renal tissues by flow cytometry and found that there was an overall increase in CD45^+^ white blood cells (Fig 4A), and particularly in CD11b^+^F4/80^+^ macrophages both at total numbers (Fig 4B and 4C) and percentages (Fig 4B and 4C). The increase in CD45^+^ total leukocytes and CD11b^+^F4/80^+^ macrophages observed in the renal tissues from DN mice relative to WT controls was significantly decreased in the renal tissues from the *Nlrc4*^-/-^ DN mice (Fig 4A–4C). In agreement with what we found by flow cytometry, we found a significant increase in F4/80^+^ macrophages in DN renal tissues relative to WT control renal tissues and that NLRC4-deficiency reduced renal macrophages by immunofluorescent staining (Fig 4D). These data suggest that NLRC4-inflammasome provides a positive signaling to recruit more kidney-infiltrating macrophages that can potentially further worsen the disease progression.

### NLRC4-deficiency leads to decreased signaling pathways related to renal pathology

**Fig 5:**
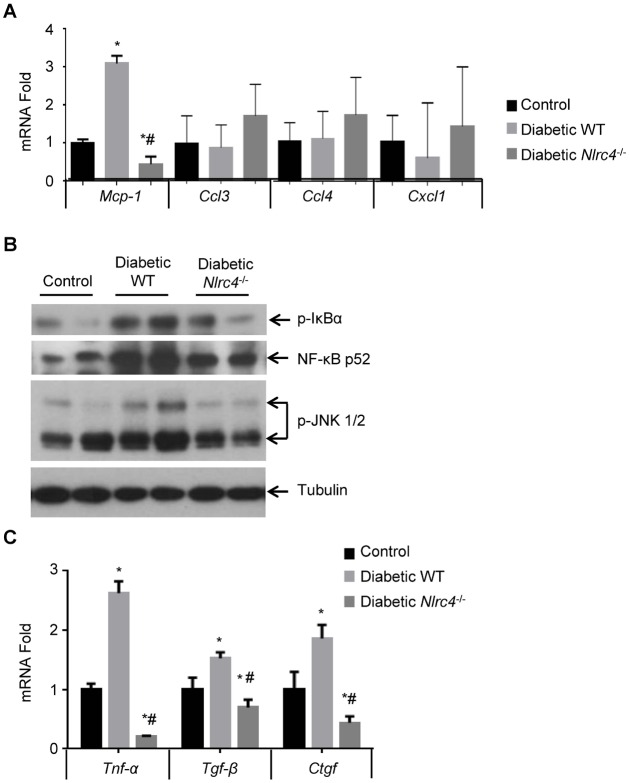
NLRC4-deficiency leads to decreased signaling pathways related to DN pathology. **(A)**. The mRNA fold change of *Mcp1*, *Ccl3*, *Ccl4*, and *Cxcl1* in the kidney tissues was determined by real-time PCR, using *Gapdh* as a reference gene, from the indicated groups. Data is presented as the average fold change compared to control ± s.e.m. n = 3–4. **(B)**. Western blot showing altered activation of different signaling pathways including NF-κB and JNK pathways in the renal tissues from different groups. Images represent two independent experiments from a total of 3 kidneys within each indicated groups. **(C)**. The mRNA fold change of *Tnfα*, *Tgfβ*, and *Ctgf* in the renal tissues was determined by real-time PCR, using *Gapdh* as a reference gene. Data is presented as the average fold change compared to controls ± s.e.m. n = 3. *, *P*<0.05 vs. control WT; #, *P* <0.05 vs. diabetic WT. n = 3.

One of the major chemotactic molecules for macrophages is monocyte chemoattractant protein-1 (MCP-1), which has been shown to play a causative role in DN models [30]. DN Patients have high levels of MCP-1 in the urine that correlates with markers of renal injury [31]. We found that renal tissues from DN mice exhibited elevated *Mcp-1* mRNA, which was reduced in renal tissues from the *Nlrc4*^-/-^ DN mice (Fig 5A), suggesting that NLRC4-inflammasome activation may influence renal infiltration of macrophages by regulating MCP-1 expression. IL-1β is known to mainly activate NF-κB and JNK signaling pathways. Using immunoblotting of renal tissues from different mice, we identified an increase in phosphorylated form of IκBα, an indicator of its degradation and downstream NF-κB activation in renal lysates from the WT DN mice compared to those from the control mice, as well as the increased expression of p52, a non-canonical NF-κB protein and the activation of JNK pathway (Fig 5B). All these signaling pathways have been shown to regulate MCP-1 expression [32, 33]. NLRC4-deficiency led to a decrease in the phosphorylation of IκBα and JNK (Fig 5B), suggesting these pathways are mediated by NLRC4-inflammasome activation. We also compared the expression of relevant cytokines including *Tnfα*, *Tgfβ* and *Ctgf* in diabetic WT and NLRC4-deficient mice by real-time PCR analyses. In agreement with the previous reports [34], we found that these cytokines were significantly increased in renal tissues from the diabetic mice (Fig 5C) in an NLRC4-dependent manner as its deficiency resulted in decreased expression of these cytokines in the renal tissues from the *Nlrc4*^-/-^ DN mice (Fig 5C). Our data suggest a correlative link between the activation of NLRC4-inflammasome, renal macrophage infiltration, and activation of signaling pathways involved in DN pathogenesis.

## Discussion

DN can be considered an inflammatory disease and several reports have demonstrated that NLRP3 inflammasome activation is associated with diabetic nephropathy [14–17]. In the current study, we establish that NLRC4-inflammasome activation is a causative reason for the disease progression of DN. We identified a potential positive feed-back regulation between NLRC4 inflammasome-driven IL-1β production and macrophage infiltration into renal tissues from the DN mice. Macrophages are likely the major cellular source for NLRC4-activation in the DN renal tissue, which in turn promotes an autocrine activation loop by inducing chemotactic molecule MCP-1 to further induce more macrophages infiltration. In support of our data, it has been shown that anakinra, an inhibitor of IL-1R1-mediated signaling, is sufficient to inhibit several pathological features related to DN in a similar mouse model of diabetes [14], further underscoring the importance to target this axis for the treatment of DN patients.

Human studies have strongly established the link between DN and the increase in plasma IL-1β level. The activation and production of IL-1β in DN patients, however, has largely been attributed to the NLRP3-inflammasome. The activation of other inflammasomes under DN has not been studied. It is known that NLRP3-inflammsome activation is associated with increased IL-1β level in the renal tissues [14]. Our study shows that NLRC4-deficiency is sufficient to reduce IL-1β levels in the renal tissues of the DN mice (Fig 3A) and significantly, suggesting that this is a major contributor for local IL-1β activation in the renal tissues. These studies suggest that the inflammasome/IL-1β axis is critical for DN progression and thus represents an attractive therapeutic target to treat DN. It is worth to note that inflammasome activation may not always contribute to T2D and the consequent DN, which is largely dependent on the activation of their downstream effectors IL-1β and/or IL-18. A recent literature showed that the activation of NLRP1 inflammasome and subsequent IL-18 production prevent obesity and metabolic syndrome, indicating that NLRP1/IL-18 axis prevents T2D and likely DN [35]. The role of AIM2 inflammasome in T2D and DN is less clear at this stage.

The NLRC4-activating signal remains unclear as the only previously identified activators of NLRC4 are bacterial flagellin and components of bacterial secretion systems [29]. Diabetic patients tend to have a higher bacterial infection rate compared to non-diabetic normal individuals. It will be interesting to know if these bacterial infections, or even sub-clinical bacterial infections, are sufficient to drive NLRC4-inflammasome activation via direct renal infection or circulating bacterial products like flagellin. Another potential explanation for NLRC4-activation in DN-renal tissues is through some other host protein that acts as a homologue to these bacterial components, thus representing a novel and as yet undefined route of activation for the NLRC4-inflammasome.

Recent studies have revealed that DN progression requires inflammation and macrophage infiltration [36, 37]. In our study, we found an increase in *Mcp-1* but not the other chemokines including Ccl3 or Ccl4 (data not shown), which was correlated with increased macrophage accumulation within the renal interstitium of the WT DN mice (Fig 4). Meanwhile, wild type diabetic mice showed glomerular hypertrophy, thickening of the glomerular basement membrane, and ECM accumulation. All of these effects were significantly alleviated in NLRC4-deficient mice (Fig 2 and Table 1), suggesting that the lack of NLRC4 inflammasome activation ameliorates kidney injury by decreasing the accumulation of macrophages.

Among the pathways that are modulated by NLRC4 inflammasome induced IL-1β, we found that NF-κB and JNK signaling pathways were increased in renal tissues of WT DN mouse in an NLRC4-depedent manner (Fig 5). NF-κB has been reported to be involved in the expression of IL-1β and Casp-1 [38] and JNK activation has been correlated with interstitial macrophage accumulation, kidney injury molecule-1 (KIM-1) expression, interstitial fibrosis, and loss of renal function in human DN [39, 40]. MCP-1, one of the major chemotactic molecules, has been shown to be regulated by both NF-κB and JNK activation [41]. We also found that the expression of *Tnfα*, *Tgfβ* and *Ctgf* mRNA, a group of cytokines known to be involved in the renal pathology of DN [42], was significantly decreased following NF-κB and JNK inactivation.

Taken together our findings provide a strong rationale to develop therapeutics for treating DN patients that are targeted against pro-inflammatory macrophage infiltration and inflammasome activation.
